# Prevalence of metabolic syndrome among urban community residents in China

**DOI:** 10.1186/1471-2458-13-599

**Published:** 2013-06-20

**Authors:** Guang-Rong Wang, Li Li, Yi-Hui Pan, Guo-Dong Tian, Wan-Long Lin, Zhe Li, Zheng-Yi Chen, You-Long Gong, George E Kikano, Kurt C Stange, Ke-Liang Ni, Nathan A Berger

**Affiliations:** 1 Zhabei District Health Bureau, Shanghai, People’s Republic of China; 2 Department of Family Medicine, School of Medicine, Case Western Reserve University, Cleveland, OH, USA; 3 Case Comprehensive Cancer Center, Case Western Reserve University/University Hospitals Case Medical Center, Cleveland, OH, USA; 4 Zhabei District Community Health Management Center, Shanghai, People’s Republic of China; 5 Zhabei District Information Management Center, Shanghai, People’s Republic of China; 6 Shibei Hospital, Zhabei District, Shanghai, People’s Republic of China; 7 School of Public Health, Fudan University, Shanghai, People’s Republic of China; 8 Departments of Epidemiology & Biostatistics and Sociology, Case Western Reserve University, Cleveland, OH, USA; 9 Department of Medicine, School of Medicine, Case Western Reserve University, Cleveland, OH, USA

**Keywords:** Metabolic syndrome, Prevalence, Population-based survey, China

## Abstract

**Background:**

Metabolic risk factors and abnormalities such as obesity and hypertension are rapidly rising among the Chinese population following China’s tremendous economic growth and widespread westernization of lifestyle in recent decades. Limited information is available about the current burden of metabolic syndrome (MetS) in China.

**Methods:**

We analyzed data on metabolic risk factors among 22,457 adults aged ≥ 32 years participating in the “Zhabei Health 2020” survey (2009–2010), a cross-sectional study of a representative sample of community residents in Zhabei District. We defined MetS using Chinese-specific cut-off points for central obesity according to consensus criteria recently endorsed by several international and national organizations in defining MetS in different populations worldwide. We used a multiple logistic regression model to assess the associations of potential risk factors with MetS.

**Results:**

The unadjusted prevalence of the MetS was 35.1% for men and 32.5% for women according to the consensus criteria for Chinese. The prevalence increased progressively from 12.1% among participants aged 32–45 years to 45.4% among those aged ≥ 75 years. Age, smoking, family history of diabetes, and education are significantly associated with risk of MetS.

**Conclusions:**

The MetS is highly prevalent and has reached epidemic proportion in Chinese urban adult community residents.

## Background

Lifestyle of the Chinese population is undergoing drastic changes following China’s rapid economic growth in recent decades. Westernization characterized by excess energy intake and sedentary lifestyle is now becoming widespread, especially among urban residents [1,2]. In parallel, metabolic risk factors, such as obesity and hypertension, are rising, and chronic diseases have surpassed infectious diseases as the leading causes of mortality and morbidity in China [2]. Indeed, cardiovascular disease is now the number one killer in China and the prevalence of type 2 diabetes mellitus has tripled during the last two decades and reached epidemic level, with almost one out of 10 Chinese (9.7%) reporting having been diagnosed with diabetes mellitus in a recent national survey [3].

The metabolic syndrome (MetS), a clustering of metabolic risk factors including hyperglycemia, dyslipidemia (elevated triglycerides, reduced high-density lipoproteins), elevated blood pressure, and obesity (central adiposity in particular), is now a well recognized clinical entity that may progress to overt diabetes mellitus and increase risks of cardiovascular disease and certain cancers [4-6]. Insulin resistance resulting from long-term energy imbalance is believed to play a critical role in the pathogenesis of MetS [7]. While there is general agreement in the medical community with regard to the components of MetS, diagnostic criteria differ among professional organizations [8-10]. A previous study based on a cross-sectional national survey of 15,540 Chinese adults aged 35–74 years in 2000–2001 reported 9.8% prevalence rate of MetS in men and 17.8% in women according to the US National Cholesterol Education Adult Treatment Panel III (NCEP ATP III) criteria, with higher rates in urban areas [11].

In an effort to harmonize the MetS diagnostic criteria, the International Diabetes Federation (IDF) Task Force on Epidemiology and Prevention and several other international and national authorities have recently issued a joint interim consensus statement that recommends population-specific cut-off points of waist circumference for central obesity in defining the MetS [12]. The waist circumference cut-off points for MetS recommended are 85 cm for Chinese men and 80 cm for women. These cut-off points are much lower than the NCEP ATP III obesity criteria (≥ 102 cm in men and ≥ 88 cm in women). Thus, the prevalence of MetS in Chinese population previously reported based on the NCEP ATP III criteria is likely to have underestimated the burden of MetS in China, [11] and the prevalence of MetS as defined by the new consensus criteria in China urban areas experiencing dramatic recent westernization of health behaviors has not been reported.

We estimated the prevalence of MetS in adults participating in a population-based study of community residents living in Zhabei District of Shanghai, China. The survey was conducted from February 2009 through February 2010, and collected data on lifestyle and metabolic risk factors on 22,757 adult participants 32 years or older. We used the joint interim consensus criteria by IDF and other organizations with Chinese specific waist circumference cut-off points in defining the obesity domain of the MetS [11]. Our study thus provides the most current estimate of the prevalence of MetS among urban residents in China. To facilitate comparison with prior studies, we also estimated the prevalence according to the modified NECP ATP III criteria [11,13].

## Methods

### Study population

The “Zhabei Health 2020” cross-sectional survey assessed lifestyle and metabolic risk factors in permanent community residents of all age selected from the entire population living in Zhabei District, Shanghai. Located in north Shanghai, Zhabei is one of the 9 urban districts of Shanghai city, with a total population of 703,526 permanent residents. Zhabei permanent residents were defined as persons registered in the Zhabei District Census Bureau who had not moved within the past five years. The survey was conducted by the Zhabei Cooperative Group on Cancer Prevention and Treatment under the auspice of Zhabei District Health Bureau.

A multistage, stratified cluster sampling method was used to select permanent residents for participation in the survey. The sampling process was first stratified by communities with the overall goal of an 8% sample of the population. The target number of participants for each of the 9 communities of Zhabei district was determined based on proportion of population residing in each community. Next, streets or villages within each community were randomly selected. Finally, buildings within the selected streets or villages were randomly selected. The survey was conducted via computer-assisted personal interview (CAPI) at participants’ home in each of the selected building. The overall response rate was 80.6%.

Residents who were 32 years and older (n=36,127) were also invited to undergo a physical examinations. Of these, 22,895 completed the CAPI survey, and 22,757 participants completed both the CAPI survey and physical examination, representing a 63.0% completion rate of the selected sample population. There were no statistically significant differences in the age and gender distributions between the respondents and the non-respondents. The survey was approved by the Institutional Review Board of Zhabei Cooperative Group of Cancer Prevention and Treatment. Analysis of the survey data was approved by the Institutional Review Board of University Hospitals Case Medical Center.

### Data collection

Data were collected via CAPI at the selected participants’ home by trained community health center staff. The CAPI collected information on demographics, personal and family medical history, and lifestyle risk factors. The interview included questions related to the diagnosis and treatment of diabetes, hypertension, hyperlipidemia, and other chronic diseases. Behavioral risk factor information such as smoking and alcohol drinking was also collected.

All participants aged 32 years and older were invited to undergo clinical physical examinations at local community health centers. Blood pressures were obtained with the participant in the seated position after 5 minute rest. Body weight and height were measured during the examination without shoes and in light hospital clothing. Waist circumference was measured at 1 cm above umbilicus, and hip circumference at the level of maximum extension of the buttocks. Participants were instructed to fast overnight before the scheduled clinical visit, and samples of blood were obtained at the clinical visit next morning. Collected samples of blood were put on wet ice and immediately shipped to the central study laboratory (Zhabei Shibei Hospital). Buffy coat, plasma and serum were aliquoted and stored at -80°C until laboratory assays could be done.

Plasma glucose was measured with a modified hexokinase enzymatic method. Lipid profile including total cholesterol, HDL-C, low density lipoprotein cholesterol (LDL-C), and triglycerides (TG) was analyzed in the Central Biochemistry Laboratory of Linfeng Community Health Center, Zhabei district.

### Metabolic syndrome (MetS) definition

We used the recently published joint interim statement endorsed by the International Diabetes Federation (IDF) Task Force and several other international and national organizations to define MetS [12]. The consensus criteria define MetS as presence of three or more of the following metabolic risk factors: 1) elevated waist circumference (population- and country-specific cut-offs: ≥ 85 cm for Chinese men and ≥ 80 cm for Chinese women); 2) elevated triglycerides ≥ 150 mg/dL (1.69 mmol/L); 3) reduced high density lipoprotein cholesterol (HDL-C) [< 40 mg/dL (1.04 mmol/L) in men, and < 50 mg/dL (1.29 mmol/L) in women]; 4) elevated blood pressure (systolic ≥ 130 mmHg and/or diastolic ≥ 85 mmHg); and 5) elevated fasting glucose ≥ 100 mg/dL (5.56 mmol/L). Individuals who reported using drug treatments for any of above medical conditions are considered met the criteria for the specific component. For comparison, we also estimated MetS using the modified NECP ATPIII criteria where HDL-C < 40 mg/dL (1.04 mmol/L) and fasting glucose ≥ 110 mg/dL (6.1 mmol/L) were used as cut-off points for both men and women to define low HDL and hyperglycemia, and waist circumference ≥ 90 cm and ≥ 80 cm were used to define central obesity, respectively for men and women [11,13].

### Statistical analysis

For all baseline characteristics, means for continuous variables and percentages for categorical variables were calculated separately for men and women, and statistical significances were examined by Student’s *t*-test and by Chi-square test for continuous and categorical variables, respectively. We calculated the distribution of participants who met the criteria for each of the 5 MetS domains defined by the joint statement or by the modified NCEP ATPIII. We then estimated the percentages of participants met 0, 1, 2, 3, 4, or 5 MetS criteria. We next estimated the total and sex specific prevalence of MetS (met at 3 out of the 5 criteria) defined by the joint statement or by the modified NCEP ATPIII. We compared the difference between males and females using Chi-square test. We also estimated the prevalence of MetS according to age groups (32–44, 45–54, 55–64, 65–74, and 75+), smoking status (yes or no), and levels of education. Finally, we estimated odds ratios (ORs) and 95% confidence intervals (95% CIs) of MetS by age, gender, family history of diabetes or coronary heart diseases (CHD), smoking status, alcohol drinking in a multiple logistic regression model. All statistical analyses were performed using R 2.12.1, and all p values were two sided with α = 0.05.

## Results

Of the 22,757 adults who had completed clinical exams, 300 reported having been diagnosed with cancer and were excluded, leaving 22,457 participants available for the final analysis. Overall, 36.4% (38.1% men, and 35.3% women) of the study participants were overweight or obese (BMI ≥ 25 kg/m2), and 57.6% men and 54.0% women were considered centrally obese according to the interim consensus waist circumference measurements for Chinese (Table 1). The self-reported prevalence of diagnosed diabetes mellitus was 9.9% in men and 9.0% in women. There was a striking gender-difference in smoking and alcohol drinking: approximately half of the male participants reported smoking cigarettes and over one third of them reported drinking alcohol, while less than 3% of females reported having either habit.

**Table 1:** Descriptive characteristics of “Health Zhabei 2020” survey adults (≥32 years)

		**Female (n=13565)**	**Male (n=8892)**	**P Value**
		**Mean (SD) or No. (%)**	**Mean (SD) or No. (%)**	
Age (years)		58.1(12.1)	59.7(12.2)	<0.001
Education level	≤Middle school	8177(60.3)	4330(48.7)	<0.001
	High school or profession school	4357(32.1)	2996(33.6)	
	≥2-year college	1022(7.5)	1553(17.5)	
	missing	9(0.1)	13(0.2)	
Smoking	Yes	334(2.5)	4509(50.7)	<0.001
	No	13078(96.4)	4265(48.0)	
	missing	153(1.1)	118(1.3)	
Alcohol Drinking	Yes	392(2.9)	3057(34.4)	<0.001
	No	13020(96.0)	5717(64.3)	
	missing	153(1.1)	118(1.3)	
Family History	CHD	1127(8.3)	699(7.9)	0.230
	Diabetes mellitus	845(6.2)	501(5.6)	0.065
Personal History	CHD	852(6.3)	435(4.9)	<0.001
	Diabetes mellitus	1227(9.0)	884(9.9)	0.025
SBP (mmHg)		125.4(15.8)	128.5 (15.2)	<0.001
DBP (mmHg)		79.1(8.9)	81.4(9.3)	<0.001
BMI (kg/m^2^)		24.1(4.4)	24.2(3.9)	0.047
	<25	8766(64.6)	5506(61.9)	<0.001
	25-30	4005(29.5)	3042(34.2)	
	>30	790(5.8)	342(3.9)	
Waist Circumference (cm)		81.0(9.7)	86.4(9.2)	<0.001
	Central obesity^a^	4521(33.3)	6989(78.6)	<0.001
Waist-Hip Ratio		0.85(0.07)	0.89(0.07)	<0.001
Fasting Glucose (mg/dL)		97.3(27.0)	99.1(30.6)	<0.001
Triglycerides (mg/dL)		132.9(106.3)	159.4(150.6)	<0.001
HDL-C (mg/dL)		61.9(65.8)	54.1(65.8)	<0.001
LDL-C (mg/dL)		116.0(34.8)	112.1(30.9)	<0.001

Abbreviations: *SBP* systolic blood pressure, *DBP* diastolic blood pressure, *BMI* body mass index, *HDL-C* high density lipid cholesterol, *LDL-C* low density lipid cholesterol.

^a^Waist ≥ 85 cm for men, ≥ 80 cm for women according to joint interim criteria.

The distribution of MetS defining risk factors is shown in Table 2. Noticeably, the prevalence rates of low HDL-C and elevated fasting glucose and/or diagnosis of diabetes were substantially higher in women and the prevalence rates of elevated fasting glucose and/or diagnosis of diabetes was substantially higher in men using the new consensus criteria as compared to that using the modified NCEP ATP III criteria. Over half of both men and women were centrally obese or having elevated blood pressure or hypertension. Table 3 shows the number and percentage of participants met 0–5 MetS criteria according to the consensus criteria or modified NCEP ATP III criteria. Approximately one-third (33.9%) of the study population met clinical diagnosis of MetS according to the consensus criteria; even after excluding those with overt diabetes mellitus or diagnosis of CHD, 29.1% of the study participants had MetS. The corresponding figures according to the modified ATP III criteria were 23.8% and 18.8% respectively.

**Table 2:** Prevalence of MetS components by gender according to the joint Interim consensus (IDF-C) and modified ATP III (ATPIII-M) criteria

	**IDF-C**^**a **^**[n(%)]**		**ATP III-M**^**b **^**[n(%)]**	
	**Female**	**Male**	**Female**	**Male**
Abdominal obesity	7328(54.0)	5120(57.6)	7328(54.0)	3195(35.9)
Elevated blood pressure or hypertension	7217(53.2)	5571(62.7)	7217(53.2)	5571(62.7)
Hypertriglyceridemia	4027(29.7)	3197(36.0)	4027(29.7)	3197(36.0)
Low HDL-C	3174(23.4)	1146(12.9)	683(5.0)	1146(12.9)
High fasting Glucose or Diabetes	3651(26.9)	2763(31.1)	2147(15.8)	1671(18.8)

^**a**^**IDF-C** (joint interim consensus criteria by IDF and other organization): Diabetes or fasting glucose ≥ 100 mg/dL; Hypertension or SBP ≥ 130 mmHg or DBP ≥ 85 mmHg; Dyslipidemia or HDL < 40 mg/dL for men and HDL < 50 mg/dL for women; Dyslipidemia or Triglycerides ≥ 150 mg/dL; Waist circumference ≥ 85 cm for men and ≥ 80 cm for women.

^**b**^**ATP III-M** (modified NCEP ATP III criteria): Diabetes or fasting glucose ≥ 110 mg/dL; Hypertension or SBP ≥ 130 mmHg or DBP ≥ 85 mmHg; Dyslipidemia or HDL < 40 mg/dL for both men and women; Dyslipidemia or Triglycerides ≥ 150 mg/dL; Waist circumference ≥ 90 cm for men and ≥ 80 cm for women.

**Table 3:** Prevalence of MetS components according to the Interim consensus (IDF-C) or the modified NCEP ATP III (ATP III-M) criteria

	**IDF-C**^**a **^**[n(%)]**		**ATP III-M**^**b **^**[n(%)]**	
**No. of Criteria met**	**DM and CHD included**	**DM and CHD excluded**	**DM and CHD included**	**DM and CHD excluded**
Met 5 criteria	686(3.1)	382(2.0)	198(0.9)	77(0.4)
Met 4 criteria	2318(10.3)	1667(8.6)	1328(5.9)	797(4.1)
Met 3 criteria	4518(20.1)	3497(18.1)	3824(17.0)	2761(14.3)
Met 2 criteria	5776(25.7)	5014(26.0)	5953(26.5)	5059(26.2)
Met 1 criteria	5386(24.0)	5026(26.1)	6502(29.0)	6031(31.3)
Met 0 criteria	3773(16.8)	3698(19.2)	4652(20.7)	4559(23.7)
total	22457	19284	22457	19284

^**a**^**IDF-C** (joint interim consensus criteria by IDF and other organization): Diabetes or fasting glucose ≥ 100 mg/dL; Hypertension or SBP ≥ 130 mmHg or DBP ≥ 85 mmHg; Dyslipidemia or HDL < 40 mg/dL for men and HDL < 50 mg/dL for women; Dyslipidemia or Triglycerides ≥ 150 mg/dL; Waist circumference ≥ 85 cm for men and ≥ 80 cm for women.

^**b**^**ATP III-M** (modified NCEP ATP III criteria): Diabetes or fasting glucose ≥ 110 mg/dL; Hypertension or SBP ≥ 130 mmHg or DBP ≥ 85mmHg; Dyslipidemia or HDL < 40 mg/dL for both men and women; Dyslipidemia or Triglycerides ≥ 150 mg/dL; Waist circumference ≥ 90cm for men and ≥ 80 cm for women.

**Figure 1:**
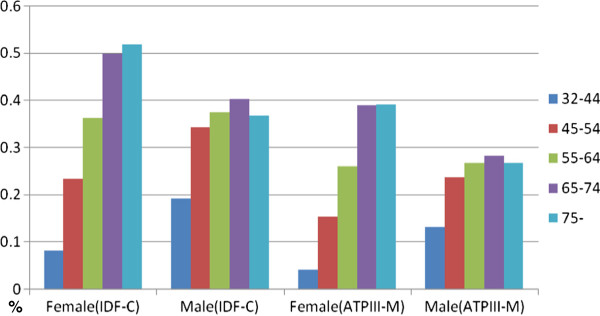
Prevalence of Metabolic Syndrome by Age and Gender, according to the Interim Consensus (IDF-C) and Modified NCEP ATP III (ATP III-M) Criteria.

**Figure 2:**
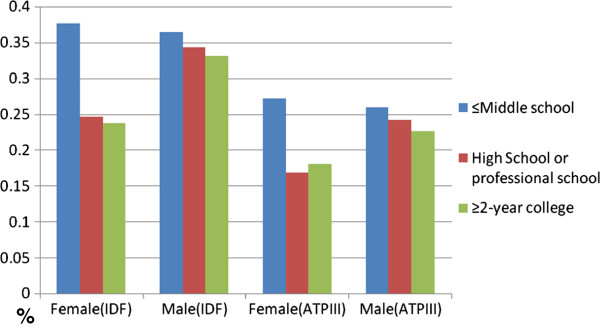
Prevalence of Metabolic Syndrome by Education and Gender, according to the Interim Consensus (IDF-C) and Modified NCEP ATP III (ATP III-M) Criteria.

Age is clearly a major determinant of MetS. The proportion of participants met 2 or more MetS criteria increased linearly with age in both men and women regardless whether the consensus or the modified NCEP ATP III criteria were used (Table 4). The prevalence of MetS increased progressively from 12.1% for those aged 32–45 years to 45.4% for those aged 75 years or older. Interestingly, the increase of the prevalence rate of MetS with age was much steeper in women as compared with men (Figure 1). The age-standardized (to the age distribution of Shanghai population reported in 2009 census) prevalence rate under joint interim census criteria was 31.5% (27.8% for women and 35.0% for men). Education level and smoking also appear to be significant correlates of MetS. In both men and women, the prevalence of MetS was appreciably higher among those with middle school or lower education as compared to those with higher levels of education (Figure 2). Among smokers, 35.0% had MetS, and 32.0% of non-smokers had MetS.

**Table 4:** Prevalence of MetS component by gender according to the Interim consensus (IDF-C) or the modified NCEP ATP III (ATP III-M) criteria

		**Met 0 criteria**		**Met 1 criteria**		**Met 2 criteria**		**Met 3 criteria**		**Met 4 criteria**		**Met 5 criteria**		
**Gender**	**Age**	**[n(%)]**		**[n(%)]**		**[n(%)]**		**[n(%)]**		**[n(%)]**		**[n(%)]**		**Total**
		**IDF-C**^**a**^	**ATPIII-M**^**b**^	**IDF-C**^**a**^	**ATPIII-M**^**b**^	**IDF-C**^**a**^	**ATPIII-M**^**b**^	**IDF-C**^**a**^	**ATPIII-M**^**b**^	**IDF-C**^**a**^	**ATPIII-M**^**b**^	**IDF-C**^**a**^	**ATPIII-M**^**b**^	
	32-44	693(46.5)	791(53.1)	456(30.6)	467(31.4)	219(14.7)	171(11.5)	86(5.8)	49(3.3)	31(2.1)	11(0.7)	4(0.3)	0(0.0)	1489
	45-54	1145(24.5)	1344(28.8)	1368(29.3)	1558(33.3)	1076(23.0)	1062(22.7)	667(14.2)	539(11.5)	321(6.9)	155(3.3)	98(2.1)	17(0.4)	4675
Female	55-64	555(14.2)	657(16.8)	915(23.5)	1082(27.7)	1019(26.0)	1150(29.5)	814(20.9)	722(18.5)	448(11.5)	257(6.6)	153(3.9)	36(0.9)	3904
	65-74	109(6.0)	121(6.6)	298(16.4)	387(21.2)	507(27.8)	605(33.2)	493(27.1)	511(28.1)	300(16.5)	174(9.6)	114(6.2)	23(1.3)	1821
	≥75	66(3.9)	87(5.2)	271(16.2)	341(20.4)	472(28.2)	594(35.4)	490(29.2)	466(27.8)	277(16.5)	166(9.9)	100(6.0)	22(1.3)	1676
	**Total**	**2568(18.9)**	**3000(22.1)**	**3308(24.4)**	**3835(28.3)**	**3293(24.3)**	**3582(26.4)**	**2550(18.8)**	**2287(16.9)**	**1377(10.1)**	**763(5.6)**	**469(3.5)**	**98(0.7)**	**13565**
	32-44	253(29.4)	344(40.0)	256(29.7)	226(26.2)	187(21.7)	179(20.8)	111(12.9)	87(10.1)	43(5.0)	20(2.3)	11(1.3)	5(0.6)	861
	45-54	409(16.9)	536(22.2)	545(22.5)	709(29.3)	636(26.3)	603(24.9)	518(21.4)	394(16.3)	253(10.5)	153(6.3)	58(2.4)	24(1.0)	2419
Male	55-64	328(11.2)	475(16.2)	678(23.1)	880(30.0)	827(28.2)	794(27.1)	684(23.4)	546(18.7)	337(11.5)	204(7.0)	76(2.6)	31(1.1)	2930
	65-74	123(8.4)	173(11.8)	304(20.6)	450(30.5)	455(30.9)	435(29.5)	378(25.6)	298(20.2)	176(11.9)	97(6.6)	38(2.6)	21(1.4)	1474
	≥75	92(7.6)	124(10.3)	295(24.4)	402(33.3)	378(31.3)	360(29.8)	277(23.0)	212(17.5)	132(10.9)	91(7.5)	34(2.8)	19(1.6)	1208
	**Total**	**1205(13.6)**	**1652(18.6)**	**2078(23.3)**	**2667(30.0)**	**2483(27.9)**	**2371(26.7)**	**1968(22.1)**	**1537(17.3)**	**941(10.6)**	**565(6.3)**	**217(2.5)**	**100(1.1)**	**8892**
**Overall**	32-44	946(40.3)	1135(48.3)	712(30.3)	693(29.5)	406(17.3)	350(14.9)	197(8.4)	136(5.8)	74(3.1)	31(1.3)	15(0.6)	5(0.2)	2350
	45-54	1554(21.9)	1880(26.5)	1913(27.0)	2267(32.0)	1712(24.1)	1665(23.5)	1185(16.7)	933(13.2)	574(8.1)	308(4.3)	156(2.2)	41(0.5)	7094
	55-64	883(12.9)	1132(16.6)	1593(23.3)	1962(28.7)	1846(27.0)	1944(28.4)	1498(21.9)	1268(18.6)	785(11.5)	461(6.7)	229(3.4)	67(1.0)	6834
	65-74	232(7.0)	294(8.9)	602(18.3)	837(25.4)	962(29.2)	1040(31.6)	871(26.4)	809(24.6)	476(14.5)	271(8.2)	152(4.6)	44(1.3)	3295
	≥75	158(5.5)	211(7.3)	566(19.6)	743(25.8)	850(29.5)	954(33.1)	767(26.6)	678(23.5)	409(14.2)	257(8.9)	134(4.6)	41(1.4)	2884
	**Total**	**3773(16.8)**	**4652(20.7)**	**5386(24.0)**	**6502(29.0)**	**5776(25.7)**	**5953(26.5)**	**4518(20.1)**	**3824(17.0)**	**2318(10.3)**	**1328(5.9)**	**686(3.1)**	**198(0.9)**	**22457**

^**a**^**IDF-C** (joint interim consensus criteria by IDF and other organization): Diabetes or fasting glucose ≥ 100 mg/dL; Hypertension or SBP ≥ 130 mmHg or DBP ≥ 85 mmHg; Dyslipidemia or HDL < 40 mg/dL for men and HDL < 50 mg/dL for women; Dyslipidemia or Triglycerides ≥ 150 mg/dL; Waist circumference ≥ 85 cm for men and ≥ 80 cm for women.

^**b**^**ATP III-M** (modified NCEP ATP III criteria): Diabetes or fasting glucose ≥ 110 mg/dL; Hypertension or SBP ≥ 130 mmHg or DBP ≥ 85 mmHg; Dyslipidemia or HDL < 40 mg/dL for both men and women; Dyslipidemia or Triglycerides ≥ 150 mg/dL; Waist circumference ≥ 90 cm for men and ≥ 80 cm for women.

Multiple logistic regression analysis revealed that age (p < 0.01), smoking (p < 0.01), family history of diabetes mellitus (p < 0.01), and low educational level (p < 0.01) were positively and statistically significantly associated with MetS. No association was found for gender, family history of CHD or alcohol drinking (data not shown).

## Discussion

In this large population-based study of community residents living in Zhabei District, Shanghai, approximately one third of the participants had MetS according to the joint interim consensus criteria recently endorsed by a number of national and international organizations [12]. As expected, the prevalence rate was lower when the modified ATPIII criteria were used to define MetS. The prevalence of MetS was slightly higher in men than that in women across all age groups, and increases linearly with age. Similarly, each of the 5 MetS defining metabolic risk factors was highly prevalent and increased with age in this urban Chinese population.

It has been well documented that MetS is highly prevalent world-wide, especially in affluent countries [14-16]. Several previous studies have reported the prevalence of MetS in Chinese population using various criteria [11,17,18]. The report by Gu et al. based on a national survey of Chinese adults aged 35–74 years conducted in 2000–2001 reported that the age-standardized prevalence of MetS was only 9.8% in men and 17.8% in women according to the NCEP ATP III criteria, and 15.1% (13.6% in men and 16.6% in women) using the modified NCEP ATP III criteria [11]. Another survey of 16,442 adults (aged ≥18 years) conducted between August and October 2005 in Beijing reported prevalence of 23.2% and 16.2% using the IDF and NCEP ATP III criteria, respectively [17]. Another study of 5,584 adults (age = 20–79 years) conducted in 2008 in Pudong New Area, a relatively affluent business district in Shanghai, reported prevalence of 28.4% for men and 35.1% for women using the modified NCEP ATP III criteria [18]. In the Shanghai Men’s Health Study by Villegas R et al., the prevalence rate for men aged 40–74 years was 29.34% according to the modified NCEP ATP III criteria for Asian populations [19]. Our present study based on a large representative sample of urban community residents in Zhabei District of Shanghai, a traditionally industrialized district of Shanghai, used the currently recommended gender-specific waist circumference cut-off points for Chinese and showed high prevalence of MetS comparable to that reported in Pudong New Area. For direct comparison, estimated age-standardized (to Shanghai 2009 census counts) prevalence of MetS among those aged 35–74 years in our study using the modified NCEP ATP III definition was 21.6% (23.7% in men and 19.4% in women), which was still considerably higher than that reported by Gu et al. Although the two surveys differed in the study design and the targeted populations sampled, the higher prevalence of MetS in our study conducted in 2009–2010 in Zhabei District of Shanghai, in conjunction with the higher prevalence reported in the study conducted in 2008 in Pudong New Area of Shanghai, as compared to the study by Gu et al. conducted in 2000–2001 may truly indicate a rapidly rising trend of MetS in a short 8–9 year span, especially among urban Chinese population. In support of this, several recent studies have also reported substantial increase of overweight/obesity and overt diabetes in China [3,20,21].

Insulin resistance is believed to be the underlying core mechanistic feature of metabolic syndrome. Obesity, abdominal adiposity in particular, plays an important role in the pathogenesis of insulin resistance and metabolic syndrome. An increasing number of comparative studies showed that at given BMIs, metabolic responses were greater in Asians as compared to Caucasians or US blacks, supporting the endorsement of ethnicity- and gender-specific cut-off points of waist for central obesity in the consensus definition of MetS [22-25]. A study comparing data from the National Health and Nutrition Survey (NHANES) III in US and from the Nutrition and Health Survey in Taiwan further showed that Chinese experienced much higher risk of hypertension, hyperglycemia, dyslipidemia, and hyperuricemia than US whites and blacks at given BMIs [24]. This discrepancy is likely due to a higher percentage of central body fat accumulation in Asians than in Caucasians at fixed BMIs [23,26,27]. In our study, even among the 1,564 men with a waist circumference of larger than 85 cm (consensus cut-off point for central obesity) but less than 90 cm (modified NCEP ATP III cut-off point), the other 4 MetS defining metabolic abnormalities are highly prevalent: 1,025 (65.5%) having elevated blood pressure or hypertension, 654 (41.8%) having hypertriglyceridemia, 526 (33.0%) having elevated fasting glucose or diabetes, and 273 (17.0%) having low HDL-C. Thus, the use of the consensus criteria with Chinese specific cut-off points for abdominal obesity is a sensible measure of the true burden of MetS in China.

The MetS is known to significantly increase the risks of diabetes and cardiovascular diseases. Even after excluding those with a known diagnosis of diabetes or CHD, the prevalence of MetS was still as high as 29.1% in our study population. At present, there is no national screening program for metabolic risk factors in China. Thus, it is conceivable that an alarmingly high proportion of the general adult urban population in China currently asymptomatic and without known diagnosis of diabetes mellitus or CHD has undiagnosed MetS and is not optimally managed for their metabolic abnormalities. These persons are at great risk of progressing to full-blown type 2 diabetes mellitus and of developing cardiovascular diseases. Indeed, a recent study has shown that the prevalence of diabetes has tripled in the recent decades in China [3]. Accumulating evidence also indicates that obesity and MetS significantly increase the risks of many cancers [28]. With the widespread urbanization and westernization following China’s rapid economic growth, there is no sign of abating of upward trend of obesity and MetS. Unchecked, it will certainly further increase the burden of type 2 diabetes mellitus and other chronic diseases in China.

There are a few limitations in our study. First, the completion rate of our study was modest at 63.0%, which could to some extent have limited the generalizability of our study results. However, comparison of age and gender distribution between the respondents and the non-respondents showed no statistically significant differences, supporting that our study sample is a good representation of the source population. Second, a cardinal feature of MetS is insulin resistance or glucose intolerance. We did not perform glucose tolerance test or directly measure circulating levels of insulin or C-peptide in this study; as such, some participants with impaired glucose tolerance but normal fasting glucose levels could have been misclassified. Nevertheless, elevated fasting glucose level is a commonly accepted criterion to define MetS.

## Conclusions

Our study clearly indicates that MetS has become an epidemic in China, and calls for immediate public health measures for the intervention on metabolic risk factors in the general Chinese population to reverse the rising trend of MetS and obesity. Studies have documented the efficacy of lifestyle and therapeutic interventions in preventing the progression of impaired glucose intolerance and MetS to overt diabetes and in reducing the risk for cardiovascular diseases [29-31]. Reduction of metabolic risk factors could be one of the most effective measures to prevent diabetes, cardiovascular diseases and certain types of cancer, and may have significant impact on the general health of the Chinese population.

## Abbreviations

MetS: Metabolic syndrome; NCEP ATPIII: National Cholesterol Education Adult Treatment Panel III; IDF: The International Diabetes Federation.

## Competing interests

All authors have completed and submitted the ICMJE Form for Disclosure of Potential Conflicts of Interest and none were reported.

## Author’ contributions

Drs. WGR and LL had full access to all of the data in the study and take responsibility for the integrity of the data and the accuracy of the data analysis. *Study concept and design*: WGR, LL, TGD, LWL, GYL, KGE, SKC, BNA. *Acquisition of data*: WGR, PYH, TGD, LWL, LZ, GYL, NKL. *Analysis and interpretation of data*: WGR, LL, TGD, LWL, CZY, KGE, SKC, BNA. *Drafting of the manuscript*: LL, CZY, BNA. *Critical revision of the manuscript for important intellectual content*: WGR, LL, PYH, TGD, LWL, CZY, KGE, SKC, BNA. *Statistical analysis*: LL, TGD, CZY. *Obtained funding*: WGR. *Administrative, technical, or material support*: WGR, LL, PYH, TGD, LWL, GYL, LZ, NKL. *Study supervision*: WGR, LL, PYH, LWL, LZ, NKL. All authors read and approved the final manuscript.

## Pre-publication history

The pre-publication history for this paper can be accessed here:

http://www.biomedcentral.com/1471-2458/13/599/prepub
